# Meeting summary: Global vaccine and immunization research forum, 2023

**DOI:** 10.1016/j.vaccine.2024.126686

**Published:** 2025-02-06

**Authors:** Birgitte Giersing, Annie X. Mo, Angela Hwang, Shahida Baqar, Kristen Earle, Andrew Ford, Carolyn Deal, Peter Dull, Martin Friede, B. Fenton Hall

**Affiliations:** a Department of Immunization, Vaccines, and Biologicals, World Health Organization, Geneva, Switzerland; b Division of Microbiology and Infectious Diseases, National Institute of Allergy and Infectious Diseases, National Institutes of Health, MSC 9825, Bethesda, MD 20892-9825, USA; c Angela Hwang Consulting, PO Box 6601, Albany, California 94706, USA; d Vaccine Development, Bill & Melinda Gates Foundation, PO Box 23350, Seattle, Washington 98102, USA

**Keywords:** GVIRF, Immunization agenda 2030, IA2030

## Abstract

At the 2023 Global Vaccine and Immunization Research Forum (GVIRF), researchers from around the world gathered in the Republic of Korea to discuss advances and opportunities in vaccines and immunization.

Many stakeholders are applying the lessons of Covid-19 to future emergencies, by advancing early-stage development of prototype vaccines to accelerate response to the next emerging infectious disease, and by building regional vaccine research, development, and manufacturing capacity to speed equitable access to vaccines in the next emergency.

Recent vaccine licensures include: respiratory syncytial virus vaccines, both for the elderly and to protect infants through maternal immunization; a new dengue virus vaccine; and licensure of Covid-19 vaccines previously marketed under emergency use authorizations. Malaria vaccine implementation is expanding and a second malaria vaccine has been recommended by the World Health Organization. In a setback for human immunodeficiency virus vaccine development, the only remaining Phase 3 trial has been discontinued.

In immunization, greater clarity is emerging on the challenges of achieving access and equity, along with strategies to address those challenges. A better understanding of behavioral and social determinants of vaccine uptake and a validated toolkit for measuring and modifying the drivers of vaccination is informing program design and service delivery, contributing to improved uptake. Implementation research, which has been essential for human papillomavirus and malaria vaccine delivery, will be critical for delivering the new respiratory syncytial virus vaccines and for many other vaccines currently in development. The growing diversity of vaccines and complexity of immunization programs are leading to greater interest in simplified regimens, combination vaccines, and other innovations to facilitate delivery.

Collaboration emerged as the unifying theme of GVIRF 2023, underscoring that the combined efforts of many contributors have enabled progress thus far, and going forward will continue to be essential to ensure equitable access to vaccines for all.

## 1 Introduction

The Global Vaccine and Immunization Research Forum (GVIRF) brings together experts in vaccine research and development (R&D), implementation science and in immunization practices to review progress and learn from their colleagues. Originally designed to track progress under the Global Vaccine Action Plan, GVIRF is now serving Immunization Agenda 2030 (IA2030) and its seventh strategic priority, Research and Innovation [1].

GVIRF is organized every two years by the World Health Organization (WHO), the US National Institute of Allergy and Infectious Diseases (NIAID), and the Bill & Melinda Gates Foundation. GVIRF 2023 was held in the Republic of Korea, drawing strength from its flourishing R&D and global health community. Poster presentations increased the diversity of research discussed and highlighted the contributions of young investigators.

This report summarizes GVIRF 2023 for a broader audience. Given the rapid pace of progress in vaccines and immunization, key developments since the meeting have been included. Meeting materials are available at https://www.technet-21.org/en/topics/global-initiatives/gvirf.

## 2 Vaccines and immunization research in context

### 2.1 Advances since GVIRF 2021

Vaccine R&D is very active, as discussed at GVIRF 2023 and summarized in the Table 1. In the last 2 years, many innovative vaccines and monoclonal antibodies for disease prevention have been prequalified, licensed, or advanced in development. These include vaccines for global diseases, such as Covid-19, respiratory syncytial virus (RSV), and tuberculosis; vaccines for regional concerns, such as dengue and malaria; and vaccines to combat future epidemics, such as for Ebola.

**Table 1:** Advances in Vaccine R&D and Innovation – highlights from GVIRF 2023

Advances	Selected Examples
**Vaccine R&D and implementation**	
Expanding implementation	Malaria vaccine
Recently licensed	Chikungunya vaccine [2] Covid-19 vaccines Dengue vaccine (second product) Malaria vaccine (second product) RSV monoclonal antibody for pediatric use RSV vaccines for the elderly and for maternal immunization
Candidates for new vaccines in the pipeline	*E. coli* vaccines Group B streptococcus vaccines for maternal immunization RSV vaccines for children *Shigella* vaccines Tuberculosis vaccines for adults and adolescents Typhoid and Paratyphi A combination vaccine
Improved products in the pipeline	Broadly protective Covid-19 vaccines “Universal” influenza vaccines Tuberculosis vaccines for infants with improved safety and efficacy
**Innovations to accelerate R&D, lower costs, and facilitate delivery**	Adjuvants for systemic and mucosal responses Heat-stable vaccines and controlled-temperature-chain labeling Micro-array patch technologies for vaccine delivery Monoclonal antibodies mRNA-based vaccines
**Initiatives and technologies to improve pandemic preparedness and access to vaccines**	100 Days Mission to Respond to Future Pandemic Threats Expanding regional vaccine manufacturing Prototype pathogens and vaccines R&D Blueprint for Epidemics Small-footprint manufacturing Vaccination across the life-course
**Initiatives to align stakeholders and inform research and innovation**	A systematic approach to understand regional vaccine R&D priorities under IA2030 Behavioral and social drivers of vaccination: tools and practical guidance for achieving high uptake Full Value of Vaccine Assessments Target Product Profiles and Preferred Product Characteristics

### 2.2 Evolution of immunization programs

Over the past 3 decades, new vaccines have been developed, introduced, and scaled up, broadening protection from disease and transforming the immunization landscape [3]. Immunization schedules have expanded to include adolescents, adults, and the elderly. The number of doses administered in each visit has increased, especially for infants. Vaccination campaigns to prevent and respond to outbreaks have further stretched immunization programs: in just one year, Covid-19 vaccination tripled the global procurement of vaccine doses [4].

Despite this progress, many children remain unprotected with basic vaccines. From 2020 to 2022, 5.5 billion people were vaccinated against Covid-19, while at the same time more than 48 million children missed getting even one dose of diphtheria-tetanus-pertussis vaccine [3,5]. Immunization programs in low-income countries have been slowest to recover from Covid-19's impact, and 33 countries have reported large measles outbreaks due to disruptions in measles vaccination [3]. Recovery from the Covid-19 pandemic and equitable access to vaccines will require strengthened immunization systems and strategies to prevent and address vaccine hesitancy [6].

Stronger immunization systems will also be needed to deliver future vaccines for seasonal administration, for high-risk populations, or for administration in heterologous prime-boost regimens. Additional needs include implementation research, to inform development and efficient delivery of novel vaccines; and innovations that simplify immunization schedules and streamline delivery, to reduce the strain on health systems and improve vaccine acceptability and uptake. Progress in these areas is discussed below.

### 2.3 Pandemic preparedness

GVIRF 2021 was held soon after the first Covid-19 vaccines received emergency use authorization. In 2023, GVIRF participants celebrated progress in vaccine R&D but were sobered by global failures to achieve rapid and equitable access to vaccines [5].

Many stakeholders are applying the lessons of Covid-19 to future emergencies. WHO member states are drafting an accord to strengthen pandemic prevention, preparedness, and response [7]. The WHO R&D Blueprint for Epidemics has identified priorities using a pathogen family approach [8]. For prototype pathogens from key viral and bacterial families, WHO will continue to lead the development of R&D roadmaps and target product profiles. NIAID has established a Pandemic Preparedness Plan to accelerate responses to new threats [9]. NIAID and the Coalition for Epidemic Preparedness Innovations (CEPI) are advancing the development of “prototype vaccines” against potential threats [10]. The 100 Days Mission is catalyzing scientific exchange for the rapid development of diagnostics, therapeutics, and vaccines, aiming to make them available within 100 days of detection of a pandemic threat [11].

In the Republic of Korea, a highly coordinated response is credited with saving more than 143,000 lives through vaccination and other public health measures [12]. The national government has invested in vaccine R&D and production capacity and is committed to strengthening international cooperation in preparation for future emergencies [13]. As a WHO global biomanufacturing training hub, the Republic of Korea is also helping to build human capacity in low- and middle-income countries [14].

GVIRF participants noted that preparedness also requires robust delivery systems and a restored consensus around the value of vaccines. They emphasized the need to “work in advance together,” with efficient collaboration, a shared vision for success, and adequate financing to build, sustain, and equitably deploy capacity for the next pandemic.

### 2.4 Regional vaccine manufacturing capacity

Vaccine manufacturing capacity is concentrated in high-income countries. This uneven distribution is associated with inequities in access, particularly during emergencies. In the African and Eastern Mediterranean regions, more than 90 % of vaccine doses are supplied by manufacturers headquartered in other regions. In 2021, while other regions with local vaccine manufacturing quickly secured Covid-19 vaccines, these two regions had the lowest access [4].

To help address this imbalance, WHO has established a technology transfer program to increase vaccine manufacturing capacity in low- and middle-income countries through collaboration and technology sharing. Due to the versatility of mRNA technologies, this program is focusing on expanding mRNA vaccine manufacturing capacity [15]. CEPI is building a network of R&D and manufacturing partners to respond rapidly to emerging threats, especially in regions with less manufacturing capacity [16]. Emerging self-contained mRNA production machinery and small-footprint manufacturing technologies have the potential to play an important role in expanding regional production capacity [17,18].

In Africa, the African Union and the Partnership for African Vaccine Manufacturing (PAVM) have called for 60 % of vaccines administered in the region to be manufactured there by 2040 [19]. In line with this vision, at least 30 manufacturing projects spanning 14 countries in Africa have been announced. Gavi, the Vaccine Alliance, is placing a higher value on supply security and supporting advance market commitments to create downstream incentives for investment [20].

These initiatives face a major challenge: market sustainability. Between emergencies, regional facilities must continue production to maintain a state of readiness. Due to high costs of entry and markets too small to achieve economies of scale, their vaccines will likely be more expensive than products from established manufacturers. Thus, sustaining regional capacity will require a willingness to pay a premium per dose for the sake of health security. In addition, regional capacity might be sustained by developing new, regionally relevant vaccines that are not available from established manufacturers [21]. To this end, WHO is leading a systematic effort to understand and inform regional R&D priorities, as a basis for setting research agendas. GVIRF speakers agreed that solving the market issue is fundamental to sustaining regional vaccine manufacturing capacity.

### 2.5 Assessing the full public health and socio-economic value of vaccines

Unlike historical vaccines that addressed widely recognized diseases, many new vaccines offer important but less conspicuous benefits. These can include: preventing disability, improving quality of life, improving educational outcomes, reducing catastrophic health expenditures, and improving productivity. Some new vaccines are intended to improve program efficiency or synergies with other health programs, or to address antimicrobial resistance. Some novel presentations are designed to improve vaccine acceptance and uptake. Making evidence-based decisions for new vaccines, therefore, requires an understanding of their full value from a societal perspective.

Full Value of Vaccine Assessments (FVVAs) are designed to meet this need [22]. At GVIRF 2023, researchers shared highlights of emerging FVVAs. For example, the FVVA for new tuberculosis vaccines predicts a 7-fold return-on-investment and cost-savings from a societal perspective in nearly all high-burden countries. Health gains will be greatest among the poor [23,24]. For group A streptococcus, vaccination could yield trillions of dollars in benefits, many times the estimated cost of vaccine development. The FVVA for measles and rubella micro-array patches estimates that they could prevent up to 37 million cases and 397,000 deaths from measles between 2030 and 2040 [25]. FVVAs are in preparation for additional pathogens, including *Shigella* [26].

## 3 Progress in research and development

### 3.1 Vaccine R&D Updates

#### 3.1.1 Respiratory syncytial virus (RSV)

RSV is the leading cause of pneumonia in infants and young children, and an important cause of lower respiratory tract illness (LRTI) in older people [27,28]. After over 5 decades of R&D, [29] multiple new products targeting RSV have gained regulatory approval as of January 2024.

For older adults, GSK's AS01_E_-adjuvanted RSV vaccine and Pfizer's unadjuvanted bivalent RSV vaccine are under review by multiple regulatory agencies and have been approved in some jurisdictions [[30], [31], [32], [33]]. Both vaccines contain the RSV F protein stabilized in the prefusion conformation.

Pfizer's RSV vaccine has also shown protection of infants through maternal vaccination at 24–36 weeks of pregnancy against medically attended severe RSV LRTI over 180 days after birth. This indication has been approved by the US Food and Drug Administration and the European Medicines Agency, and is under review by multiple other agencies [34].

Monoclonal antibodies (mAbs) to protect infants from RSV were first approved in 1998, but their use has been limited due to high cost and short duration of protection [35]. A new mAb addresses these limitations. Nirsevimab targets a prefusion epitope on the F protein and contains mutations to extend its half-life. It has shown efficacy against RSV-associated LRTI over 5 months when administered as a single dose in advance of the RSV season. Researchers estimate that one LRTI hospitalization would be prevented for every 53 infants who receive nirsevimab [36]. As of September 2023, nirsevimab is under review by multiple regulatory agencies and has been approved in some jurisdictions [37].

Preventing RSV disease would have the greatest benefit in low- and middle-income countries, where more than 97 % of RSV-attributable deaths occur [28]. To facilitate uptake in these settings, Pfizer is preparing to manufacture multi-dose vials of its RSV vaccine for maternal immunization, with pricing commitments for Gavi-eligible countries. An impact study to assess the public health benefits of RSV prevention is being planned.

#### 3.1.2 Dengue

With a burden of up to 96 million symptomatic infections and 36,000 deaths each year, dengue is a disease of high morbidity but relatively low mortality [38]. Four serotypes of the dengue virus can cause the full spectrum of disease, from asymptomatic infections to severe dengue and death. The risk of severe dengue is greatest when an individual is infected sequentially with viruses of different serotypes. These immunological interactions between serotypes create substantial challenges for dengue vaccine development. Because the efficacy of a dengue vaccine depends on the vaccinated person's prior exposure to each type of the dengue virus and on the type-specificity of the antibody responses generated by the vaccine, clinical trials should consider endpoints stratified by baseline serostatus and serotypes, and trials need to be conducted for 3 to 5 years [38].

The first dengue vaccine was licensed in 2015 but has seen little uptake because WHO recommends that its use be limited to dengue seropositive individuals to avoid increasing the risk of severe dengue [38]. A second vaccine has been approved by multiple regulatory agencies starting in 2022 and WHO has recommended its introduction in settings with high dengue burden and transmission intensity [39]. A third dengue vaccine is currently being evaluated in a Phase 3 trial [40].

#### 3.1.3 Malaria

Malaria caused an estimated 241 million cases and 627,000 deaths globally in 2020 [41]. The first malaria vaccine, GlaxoSmithKline's RTS,S/AS01, has been prequalified for procurement by United Nations agencies. WHO has issued recommendations for its use, informed in part by pilot introductions in Ghana, Kenya, and Malawi [41]. Vaccination has been expanded throughout the pilot areas and as of mid-2023, more than 5 million doses have been delivered through the childhood immunization programs in the pilot countries. Gavi-supported introductions in 9 additional countries will begin in early 2024 [42]. Research questions relating to RTS,S implementation include how to increase uptake of the fourth dose and how to conduct seasonal malaria vaccination for increased vaccine efficacy and impact [43,44].

Because demand for the vaccine is very high, initial allocations have been guided by a framework for allocation [45]. To increase manufacturing capacity, GlaxoSmithKline will be transferring production to Bharat Biotech. As of October 2023, a second malaria vaccine, R21/Matrix-M, has received approval from regulators in Ghana, Nigeria, and Burkina Faso, and has been recommended by WHO [46,47]. Availability of this vaccine will further improve supply.

Malaria vaccines with higher efficacy and longer duration of protection are still needed. In the medium term, the current vaccines might be combined with antigens targeting the blood stage of the parasite lifecycle. Such multistage vaccines could provide synergistic protection with modest increases in cost of goods [48]. Longer-term prospects in early development include transmission blocking vaccines, vaccines against other plasmodium species, and vaccines that protect against malaria in pregnancy.

#### 3.1.4 Tuberculosis

In 2023, an estimated 10.8 million people developed tuberculosis and 1.25 million people died of tuberculosis. Of those, 161,000 deaths were among people living with human immunodeficiency virus (HIV) [49]. The currently available vaccine, Bacille Calmette-Guérin (BCG), prevents severe tuberculosis in young children but has limited efficacy in preventing pulmonary tuberculosis in adolescents and adults, who are the main transmitters of the pathogen.

As of August 2024, 15 tuberculosis vaccine candidates were in clinical development. These included MTBVAC and VPM1002, live vaccines designed to improve on the safety, efficacy and manufacturability of BCG. Both candidates are in Phase 3 studies in infants (ClinicalTrials.gov NCT04975178 and NCT04351685), and VPM1002 is also in Phase 3 trials in household contacts of people with tuberculosis and a Phase 3 prevention of recurrence study in adults (NCT03152903). A Phase 3 study for the most advanced protein vaccine candidate, M72/AS01_E_, has begun and will be conducted across seven high burden countries [50].

Additional efforts to advance new tuberculosis vaccines include the development of global roadmaps for tuberculosis vaccine R&D, a global framework to prepare for country introduction, guidance on evidence needs for policy recommendations, and the FVVA discussed above [[51], [52], [53], [54]].

#### 3.1.5 Influenza

Seasonal influenza causes an estimated 1 billion illnesses and 290,000 to 650,000 deaths annually [55]. Vaccines that elicit durable protection against all or multiple influenza viruses would help reduce the burden of seasonal influenza and improve preparedness for future pandemics. R&D for broadly protective influenza vaccines is very active: as of October 2024, 225 candidates are in development, including 40 candidates in clinical testing. Four candidates are combination vaccines targeting both influenza and Covid-19 [56].

Recent highlights include a Phase 3 trial of a Matrix-M-adjuvanted quadrivalent nanoparticle influenza vaccine, which showed enhanced humoral and cellular immune responses compared to a licensed, inactivated influenza vaccine [57]. Additionally, a multivalent mRNA vaccine encoding antigens from 20 influenza strains protected mice against challenge with matched and mismatched strains, showing the potential usefulness of mRNA approaches for universal influenza vaccine development [58].

#### 3.1.6 Human immunodeficiency virus (HIV)

WHO estimates that 1.3 million people acquired HIV and 630,000 people died of HIV in 2023 [59]. HIV vaccine development has been extremely challenging due to the complex biology of HIV-host interactions. Most recently, Mosaico, the only ongoing Phase 3 HIV vaccine trial was discontinued [60]. This trial found that a regimen of adenovirus-vectored mosaic HIV proteins in combination with adjuvanted Clade C and mosaic gp140 was generally safe and well tolerated, but not effective at preventing HIV acquisition.

Current HIV vaccine-related studies include sequential immunization to induce the production of broadly neutralizing antibodies and experimental medicine approaches to understand the immune response to HIV, design immunogens, and explore potential correlates of protection [[61], [62], [63]]. In addition, broadly neutralizing mAbs are being evaluated for passive prevention of infection for several months at a time [64]. Such mAbs must compare favorably to existing antivirals in safety, efficacy, dosing schedule, and cost if they are to prove beneficial [65].

#### 3.1.7 Group B streptococcus (GBS)

GBS is a common commensal that can ascend the genital tract during pregnancy and cause maternal sepsis, stillbirth and preterm birth, and infant sepsis and meningitis. Globally, GBS causes 46,200 stillbirths and 91,900 infant deaths each year [66]. Intrapartum antibiotic prophylaxis can prevent early onset sepsis in infants but is difficult to implement in low-resource settings, where the burden of GBS is greatest. Moreover, it is unlikely to prevent GBS infections in infants after the first day of life, or to prevent GBS-associated stillbirths or preterm births.

To help address these needs, WHO has defined preferred product characteristics for vaccines to prevent GBS-related disease. These vaccines would be administered in pregnancy and protect infants through placental antibody transfer [67]. Candidates in clinical development include protein subunits and conjugated polysaccharide vaccines [68,69]. Experts anticipate that trials evaluating efficacy against invasive GBS disease will not be feasible, and have proposed strategies to accelerate licensure based on correlates of protection [70].

### 3.2 Special populations

**Pregnant persons**. Despite changes in immune responses during pregnancy, pregnant and non-pregnant people can respond similarly to vaccination [71]. WHO recommends immunization during pregnancy to protect mothers, fetuses, and infants from Covid-19, influenza, pertussis, and tetanus [72,73]. An RSV vaccine for maternal immunization has been approved [34] and GBS vaccines for maternal immunization are in development.

Perspectives on vaccination during pregnancy have been shifting. In the past, many trials have excluded pregnant persons to avoid any risk of harm. A more balanced view is emerging that also considers disease risks and the ethical obligation to enable evidence-based decisions, including in pregnancy. This shift is not complete: during the Covid-19 pandemic, exclusion of pregnant persons from early vaccine trials contributed to evidence gaps and unclear guidance for vaccination during pregnancy [74]. Speakers at GVIRF 2023 emphasized that the needs of pregnant persons and their infants should be considered when designing vaccine trials, and indeed, throughout clinical development.

**The elderly**. Immune cell functions decline with age, and comorbidities such as diabetes can also weaken immune defenses. In the Covid-19 pandemic, increasing age was recognized as a risk factor for severe disease. Greater stimulation, for example with higher antigen content, adjuvants, or booster doses, can increase immune responses. Viral-vectored and mRNA vaccines may be more immunogenic among the elderly [75].

**People living with HIV (PLWH)**. Among PLWH, chronic inflammation can accelerate aging of the immune system. Immune responses to vaccines are generally good, but may differ from those seen in the general population [75]. As PLWH on antiretroviral therapy live longer, the combined effects of chronic HIV infection and aging on the immune system become increasingly important.

### 3.3 Mucosal immunity

R&D for mucosal vaccines is intensifying because they are seen as having a greater potential than parenteral vaccines to block infection through mucosal surfaces. Mucosal immunity is distinct from systemic immunity: circulating antibodies typically do not confer immunity at mucosal surfaces, and mucosal immunity is short-lived compared to systemic immunity [76].

Multiple companies are adapting injectable Covid-19 vaccines to mucosal administration [77]. CanSino has developed an aerosolized form of their adenovirus-vectored vaccine originally licensed for intramuscular administration. The aerosol has been approved in China as a Covid-19 vaccine booster [78]. Moderna is developing an intranasal form of their Covid-19 mRNA vaccine. In preclinical studies, the intranasal form protects hamsters from SARS-CoV-2 viral challenge [79]. Castlevax is developing Covid-19 vaccines using a live-attenuated Newcastle disease virus platform designed for low-cost, local vaccine manufacture. The injected form of the vaccine is currently in Phase 2/3 trials and the intranasal version is currently in a Phase 1 trial. (NCT05181709) Since intranasal administration of a toxin-adjuvanted influenza vaccine has been associated with Bell's palsy [80], clinical development for these vaccines will need to show that the benefits of intranasal administration outweigh the potential risks.

### 3.4 Vaccine platforms

**mRNA**. A review of mRNA vaccine R&D presented at GVIRF 2023 described how companies are applying mRNA technology to global health targets, including both endemic diseases and epidemic threats, and to therapeutic indications. Researchers are exploring alternative routes of administration (beyond intramuscular injection) and aiming to establish a platform for mRNA intranasal administration that gives both systemic and mucosal immunity.

**Virus-like particles** (VLPs) are the basis for many existing vaccines, including human papillomavirus, hepatitis B, malaria, and Covid-19. Additional applications are being developed, including broadly protective Covid-19 vaccines, VLPs for mucosal administration, and mRNA-VLP combination vaccines. Recent advances in structure-based immunogen design have enabled greater control over the size, shape, composition, location, and orientation of proteins displayed on VLPs, and promise to further improve VLP vaccine effectiveness [81]. Because they can display multiple antigens and be manufactured with high yields, VLPs could also improve access to new vaccines.

**Viral vectors.** Use of viral-vectored vaccines has expanded due to the use of adenovirus-vectored vaccines against Covid-19 [82]. Advantages of this platform include: established global production capacity; an ability to elicit cellular, humoral, and mucosal immunity; and better thermostability than current mRNA vaccines. Non-replicating viral vectors can be used in immunosuppressed individuals. Disadvantages include the potential role of pre-existing or induced anti-vector immunity, however this has not yet been observed. Development of viral vector-based approaches continues with refinement of vector backbones and evaluation of alternative delivery routes such as by aerosol [82].

### 3.5 Improving deliverability

The Vaccine Innovation Prioritization Strategy (VIPS) is a Gavi-led collaboration to improve coverage and equity through innovations that enhance deliverability. VIPS priorities include micro-array patches (MAPs) and heat-stable and controlled-temperature-chain (CTC)-qualified vaccines.

**MAPs** are small, single-dose devices containing hundreds or thousands of vaccine-coated microprojections that deliver vaccines into the surface of the skin. MAPs are intended to lower delivery costs and improve ease of use, thermostability, and acceptability, thereby increasing access. VIPS is addressing challenges for MAPs, including technical and regulatory hurdles, high upfront costs, and demand uncertainty [83]. For measles-rubella MAPs, an FVVA has been developed [25] and safety and immunogenicity data in young children were announced in mid-2023 [84]. A pivotal phase 3 trial is in preparation.

**CTC**. While most vaccines must be kept at 2 to 8 °C to maintain potency, some can remain stable at higher temperatures. CTC qualification enables these more stable vaccines to be stored at ambient temperatures (up to at least 40 °C) for a limited time (up to at least 3 days) once, just prior to administration. Vaccines for meningitis A, human papillomavirus, cholera, and typhoid have been CTC-qualified, but are not yet widely used in CTC. To inform product developers, VIPS is working with immunization programs to prioritize vaccine thermostability improvements.

### 3.6 Optimizing vaccine regimens

The increasing complexity of vaccination schedules and number of injections given per visit have heightened interest in vaccine regimens that can improve vaccine acceptability, effectiveness, or duration of protection.

Changes to vaccine doses and dosing regimens can improve efficacy and duration of protection but can also have important programmatic implications. Higher doses generally give higher immunogenicity, but they can be more costly to manufacture and can also be more reactogenic than lower doses. Regimens with more doses can be more immunogenic, but increasing the number of doses also increases delivery costs and can contribute to lower adherence. Furthermore, when a vaccine is in limited supply, vaccinating more people with fewer doses could yield greater impact [85]. Longer intervals between doses generally give higher immunogenicity but leave longer periods of risk before achieving full protection. Moreover, adherence can decline for later doses [86]. In infants, older age and lower maternal antibody levels are associated with stronger immune responses; the optimal timing of infant vaccination, however, depends on the risk of disease, maturity of the infant immune system, maternal antibody levels, and coverage in antenatal immunization programs and the antigens and carrier proteins they administer [87]. For all these reasons, changes to vaccine doses and regimens pose difficult but important tradeoff questions. The evolution of human papillomavirus (HPV) vaccine regimens illustrates the value of working through such questions: in 2022, WHO issued updated recommendations supportive of single-dose HPV vaccination for persons aged 9–20 years, facilitating the expansion of HPV vaccination worldwide [88,89].

Combination vaccines can add antigens to vaccination schedules without increasing the number of injections, and many combinations are currently in use or under development. Newer mRNA technologies can improve the feasibility of developing combination vaccines, but realizing their potential will also require access to intellectual property for each vaccine component. Demand from countries can encourage developers, funders, and policy makers to overcome intellectual property hurdles.

## 4 Progress in access and implementation

### 4.1 Zero-Dose Children

The number of zero-dose children—those who do not receive any vaccines—reflects levels of inequity. In 2022, immunization programs were recovering from the Covid-19 pandemic, and the number of zero-dose children improved from 18.1 million in 2021 to 14.3 million. Nevertheless, the number of zero-dose children remained higher than pre-pandemic levels, showing that recovery remained incomplete [3].

Zero-dose children typically live in communities subject to multiple deprivations. Their communities can be remote and rural, the urban poor, affected by conflict, or constrained by gender barriers. They often lack clean water, adequate nutrition, and access to education. A lack of basic social services often fosters cynicism and mistrust of authorities [90]. Because of the diversity of challenges, more resources and effort are required to reach these communities.

To address these inequities, Gavi has launched an Equity Accelerator Fund to help countries identify and develop strategies to reach zero-dose children [91]. WorldPop has created a vulnerability index that combines data on social factors associated with zero-dose status and gridded population datasets, to identify communities likely to have high numbers of zero-dose children. This index can help focus resources on the communities where more zero-dose children are likely to live [92]. Increasing mobile phone use across the world has created opportunities to apply low-cost digital health tools in immunization programs. For example, electronic immunization registries can be linked with personalized text message reminders for vaccination [93]. Data visualizations and dashboards developed for Covid-19 could be translated to routine immunization monitoring activities.

GVIRF 2023 participants emphasized that reaching zero-dose children requires addressing the structural barriers to immunization, as described in the Reaching Every District strategy [94]. Rather than focusing just on technology solutions, services should be multi-sectoral and tailored to community needs and priorities.

### 4.2 Implementation research

Implementation research has been defined as, “The scientific study of methods to promote the systematic uptake of research findings into routine practice.” [95] Implementation research is especially crucial for new vaccines that target age groups or populations who are not routinely vaccinated.

For example, pilot implementations of the RTS,S malaria vaccine have provided data on vaccine safety, effectiveness, uptake, and delivery costs in routine vaccination of children older than 5 months, informing WHO recommendations for use of the vaccine [41]. More research is needed to learn how to achieve high coverage in countries with low routine immunization coverage, how to reduce the cost of mass campaigns, how to reach nomadic populations, and how to vaccinate in insecurity-prone areas.

Similarly, HPV vaccine delivery has benefitted from implementation research on ways to reach 9-to-14-year-old girls and on cultural and social norms relating to vaccinating this population. Current questions include how to integrate HPV with other adolescent health services, how to reach out-of-school girls, and how to reduce program costs [96].

Implementation research will also be essential for new products to protect infants from RSV, which are administered during pregnancy or at birth [34,36]. In delivering tetanus vaccines to pregnant persons and birth doses of other vaccines to newborns, many lower-income countries have gained experience in coordinating antenatal care and immunization services to reach these populations [97]. Implementation research will be needed to learn: how to increase coverage, how to accommodate new products with exacting delivery requirements, and how to build awareness of RSV and acceptance of these new interventions.

### 4.3 Understanding and addressing low uptake

**Figure 1:**
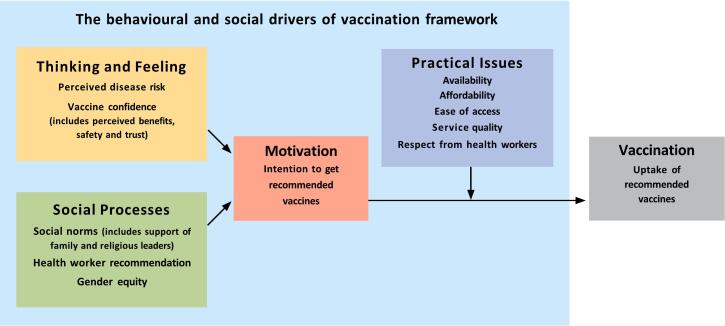
Behavioral and social drivers of vaccination – a measurement framework.

Many factors influence whether a person is vaccinated. To help programs to understand and address the reasons for low uptake, a new framework of behavioral and social drivers (BeSD) of vaccination identifies 4 domains that can be measured to inform program improvements, and modified to increase uptake (Figure 1) [98]. The framework is accompanied by tools and guidance to help programs collect data, identify interventions, plan effectively, and track progress.

At GVIRF 2023, two examples illustrated the value of the BeSD perspective. In the first example, UNICEF explored the drivers of Covid-19 vaccination in 23 countries in the Middle East and North Africa. Through behavioral surveys, they found that intention to be vaccinated was high, but many were not yet vaccinated due to service-related and structural issues. These results guided the design of interventions to lower the barriers to vaccination. For example, in Sudan, offering vaccination at places and events that women frequented and providing “women only” vaccination sites resulted in about a 10 % increase in uptake among women [99].

In the second example, commercial social listening tools trained using vaccine-related word prompts were used in Nigeria to monitor perspectives on the Covid-19 vaccination program, including on vaccine preference, locations of vaccination sites, vaccine availability, and government policies. Data were analyzed weekly and helped to inform where vaccinators were deployed and messages to tackle disinformation and raise public confidence [100].

Lessons from these studies underscored that people and equity should not be an afterthought but the starting point, and that community engagement is needed from the earliest stages of implementation. Communication and educational interventions should be tailored to disadvantaged and at-risk populations, and should be combined with high-quality service delivery that meets the needs of communities [101].

## 5 Moving forward together

Collaboration emerged as the unifying theme of GVIRF 2023. Covid-19 has shown that strong partnerships can achieve wonders in mobilizing finances, speeding up vaccine R&D and access, and saving lives. It has also shown that gaps in solidarity can expand inequities and heighten risks for all.

As highlighted by IA2030, [102] collaboration is needed to address longstanding public health needs amidst the increasing and interconnected challenges of climate change, scarcity, and conflict. Partnership is needed to ensure that vaccines address regional and country priorities and meet the needs of health systems, and to build motivation for vaccination. Equitable access to vaccines will require shared commitments to incentivizing, building, and sustaining regional manufacturing capacity. And if we do not collaborate to address these challenges, we will find ourselves repeating the failures of the past.

In summary, one speaker quoted Henry Ford who said, “Coming together is a beginning, keeping together is progress, and working together is success.” In two years, GVIRF will bring the vaccines and immunization research community together again to foster collaboration and shared success.

## Funding

This forum was supported in part by a NIAID cooperative agreement [grant number U01AI139547], grants from the Bill & Melinda Gates Foundation [grant numbers OPP1128274 and INV-005318], and an extraordinary grant from the International Vaccine Institute. Under the grant conditions of the Bill & Melinda Gates Foundation, a Creative Commons Attribution 4.0 Generic License has already been assigned to the Author Accepted Manuscript version that might arise from this submission.

## Disclaimer

This report is a summary of the GVIRF 2023 meeting and does not necessarily reflect the views of the Bill & Melinda Gates Foundation, the US National Institutes of Health (NIH)/NIAID, and WHO. It should not be construed as an official Bill & Melinda Gates Foundation, NIH/NIAID, or WHO position, policy, or decision unless so designated by other documentation. No official endorsement should be made.

## CRediT authorship contribution statement

**Birgitte Giersing:** Writing – review & editing, Writing – original draft. **Annie X. Mo:** Writing – review & editing, Writing – original draft. **Angela Hwang:** Writing – review & editing, Writing – original draft. **Shahida Baqar:** Writing – review & editing. **Kristen Earle:** Writing – review & editing. **Andrew Ford:** Writing – review & editing. **Carolyn Deal:** Writing – review & editing. **Peter Dull:** Writing – review & editing. **Martin Friede:** Writing – review & editing. **B. Fenton Hall:** Writing – review & editing.

## Declaration of competing interest

The authors declare the following financial interests/personal relationships which may be considered as potential competing interests: Birgitte Giersing and Martin Friede report financial support was provided by Bill & Melinda Gates Foundation. Angela Hwang reports financial support and travel were provided by Bill & Melinda Gates Foundation. Annie X. Mo, Shahida Baqar, Andrew Ford, Carolyn Deal, and B. Fenton Hall report relationships with the National Institute of Allergy and Infectious Diseases that include: employment and travel reimbursement. Birgitte Giersing and Martin Friede report relationships with the World Health Organization that include: employment and travel reimbursement. Kristen Earle and Peter Dull report relationships with the Bill & Melinda Gates Foundation that include: employment and travel reimbursement. Angela Hwang reports a relationship with World Health Organization that includes: consulting or advisory and travel reimbursement. WHO receives grant funding related to developing guidance for a number of vaccines referred to in the manuscript. (BG and MF) NIAID is a leading supporter of vaccine R&D, however the co-authors employed by NIAID have no conflicts of interest in their routine course of employment. (AXM, SB, AF, CD, BFH).

## Data Availability

No data was used for the research described in the article.
